# MULTIMORBIDITY RESILIENCE IN COMMUNITY-RESIDING OLDER ADULTS: MEASUREMENT AND HEALTH OUTCOMES

**DOI:** 10.1093/geroni/igz038.3361

**Published:** 2019-11-08

**Authors:** Yingzhi Xu, Eleanor McConnell, Tingzhong (Michelle) Xue, Kirsten Corazzini

**Affiliations:** 1 Duke University, Durham, North Carolina, United States; 2 University of Maryland School of Nursing, Baltimore, Maryland, United States

## Abstract

Multimorbidity is widespread, costly, and associated with a range of deleterious outcomes; it affects an estimated 67-80% of older adults. This study tests the validity of a multimorbidity resilience index developed in a Canadian sample of older adults by Wister et al., (2018), with a U.S.-based sample, using National Social Life, Health, and Aging Project (NSHAP) data, and draws upon the index to investigate the effects of resilience on outcomes over time. We mapped Wister et al.’s (2018) index to NSHAP measures, and assessed cross-sectional associations with health outcomes, using logistic regression. To assess the effects of resilience on health outcomes over time, we estimated mixed models of the relationships between resilience on outcomes over a 5-year interval. Total resilience was consistently associated with improved outcomes, including pain level (OR=.51, CI .41-.64); reduced utilization (OR=.45, CI .33-.60); improved mental health (OR=9.13, CI 6.20-13.44); self-rated physical health (OR=6.97, CI 4.76 10.19); and sleep quality (OR=3.66, CI 2.76-4.86). Longitudinal model results indicate change in multimorbidity resilience and number of chronic diseases predict (α=.001) pain level and self-rated physical health. Effects were moderated by socio-demographic factors. Our findings validate Wister et al.’s (2018) resilience index in a U.S. sample, supporting the importance of this measure to capture core components of older adults’ capacity to sustain well-being in the context of living with multiple, chronic conditions. Results from the longitudinal models provide beginning insights into the effects of resilience on symptom experience and perceived health over time, highlighting potential levers for change.

